# Identification of Novel CELSR1 Mutations in Spina Bifida

**DOI:** 10.1371/journal.pone.0092207

**Published:** 2014-03-14

**Authors:** Yunping Lei, Huiping Zhu, Wei Yang, M. Elizabeth Ross, Gary M. Shaw, Richard H. Finnell

**Affiliations:** 1 Dell Pediatric Research Institute, Department of Nutritional Sciences, The University of Texas at Austin, Austin, Texas, United States of America; 2 Department of Chemistry, College of Natural Sciences, The University of Texas at Austin, Austin, Texas, United States of America; 3 Department of Pediatrics, Division of Neonatology, Stanford University School of Medicine, Stanford, California, United States of America; 4 Center for Neurogenetics, Brain and Mind Research Institute, Weill Cornell Medical College, New York, New York, United States of America; University of Bonn, Institut of experimental hematology and transfusion medicine, Germany

## Abstract

Spina bifida is one of the most common neural tube defects (NTDs) with a complex etiology. Variants in planar cell polarity (PCP) genes have been associated with NTDs including spina bifida in both animal models and human cohorts. In this study, we sequenced all exons of CELSR1 in 192 spina bifida patients from a California population to determine the contribution of CELSR1 mutations in the studied population. Novel and rare variants identified in these patients were subsequently genotyped in 190 ethnically matched control individuals. Six missense mutations not found in controls were predicted to be deleterious by both SIFT and PolyPhen. Two TG dinucleotide repeat variants were individually detected in 2 spina bifida patients but not detected in controls. In vitro functional analysis showed that the two TG dinucleotide repeat variants not only changed subcellular localization of the CELSR1 protein, but also impaired the physical association between CELSR1 and VANGL2, and thus diminished the ability to recruit VANGL2 for cell-cell contact. In total, 3% of our spina bifida patients carry deleterious or predicted to be deleterious CELSR1 mutations. Our findings suggest that CELSR1 mutations contribute to the risk of spina bifida in a cohort of spina bifida patients from California.

## Introduction

Neural tube defects (NTDs) are among the most severe and common of all human birth defects. The most frequent types of NTDs are spina bifida and anencephaly. The etiology of NTDs is complex and involves both environmental and genetic factors. Periconceptional folic acid supplementation reduces 50% to 70% of newborn NTDs [1]; however, the mechanisms underlying this protective effect remain unclear. In terms of genetic underpinnings, monozygotic twinning and single gene disorders have long been associated with increased risks of NTDs [2]. Numerous exploratory candidate gene studies have highlighted a variety of biological pathways such as the folate and one carbon metabolism and transport [3], DNA repair [4], retinoic acid receptors [5], and the planar cell polarity (PCP) signaling network [6].

The PCP pathway controls the polarity of cells within the plane of epithelium in both vertebrates and invertebrates. The PCP genes, including *frizzled*, *dishevelled*, *vangl*, *flamingo (Celsr)*, *prickle* and *diego*, were initially identified in *Drosophila*, and are highly conserved throughout evolution [7]. PCP signaling is required for the initiation of neural tube closure in higher vertebrates [8]. In mice, mutations in *Vangl2*, *Celsr1*, *Dishevelled* and *Frizzled* result in the NTD known as craniorachischisis [9]. In humans, mutations in *FRIZZLED6* [10], *DISHEVELLED* [11], *VANGL* [12], [13] *SCRIB* [14] and *PRICKLE1* [15] have been associated with NTDs.

CELSR1 is well known for regulating the establishment and maintenance of planar cell polarity. During mitosis, CELSR1 recruits VANGL2 and FZD6 to endosomes. Following mitosis, CELSR1, VANGL2 and FZD6 are recycled to the cell surface to re-establish cell polarity [16]. In *Drosophila*, CELSR1 mediates homotypic interaction between adjacent cells and transmits instructive PCP signals. In zebrafish, the knocking down of *Celsr1* produced convergent extension (CE) defects [17]. In mice, *Celsr1* mutants exhibited craniorachischisis, the most severe form of NTDs [18]. In humans, functional *CELSR1* single nucletotide variants (SNVs) have been identified in fetuses with craniorachischisis, and predicted-to-be-deleterious *CELSR1* SNVs have been detected in a few cases with NTDs or caudal agenesis [19], [20]. However, the contribution of *CELSR1* mutations in the etiology of spina bifida is still unknown. In this study, we investigated the *CELSR1* coding region sequence among a cohort of spina bifida infants born in California by Sanger sequencing. Further, we conducted *in vitro* functional analyses to validate the functional effect of the identified mutations.

## Materials and Methods

### Ethics statement

The approval process includes detailed review by the State of California Committee for the Protection of Human Subjects (the primary IRB). All samples were obtained with approval from the State of California Health and Welfare Agency Committee for the Protection of Human Subjects.

### Human subjects

Data were obtained from a population-based case–control study conducted by the California Birth Defects Monitoring Program (CBDMP). The CBDMP is an active, population-based surveillance system for collecting information on infants and fetuses with congenital malformations, which has been described elsewhere [21]. Included for study were 192 infants with isolated spina bifida and without other major birth defects (cases) and 190 non-malformed infants (controls). Cases randomly selected from all live born infants with spina bifida and a random sample of non-malformed infants were ascertained by the CBDMP corresponding to birth years 1983–1999. Among the 192 spina bifida cases, 82 are White NonHispanic, 54 are native US born Hispanics and 56 are foreign born Hispanics. Among the 190 controls, 81 are White NonHispanic, 54 are US born Hispanics and 55 are foreign born Hispanics. All of the 192 spina bifida included in this study are cases of myelomeningocele. The case and control infants were linked to their newborn screening bloodspots, which served as the source of the gDNA used in these studies. Bloodspots are collected on all newborns in California for genetic testing purposes by the State of California. The State retains the residual, unused portion of the bloodspot and makes these bloodspots available to approved researchers.

### DNA sequencing

Genomic DNA was extracted using the Puregene DNA Extraction Kit (Qiagen, Valencia, CA) and amplified using the GenomiPhi Kit (GE Healthcare). Coding exons and flanking exon-intron regions of the human *CELSR1* gene (NM_014246) were amplified by polymerase chain reactions (PCR) from the whole genome amplification (WGA) product. Primer sequences are available upon request. The PCR products were sequenced using the Prism Bigdye Terminator Kit (v3) on an ABI 3730XL DNA analyzer (Life Technologies, Carlsbad, CA). Both case and control samples were sequenced with either a specific forward or reverse primer. Sequencing results were analyzed using the Mutation Surveyor software V4.0.7 (Softgenetics, Stage College, PA). Detected mutations were subsequently confirmed by a second round of whole genome amplification, PCR and sequencing analysis. GenomiPhi Kit takes advantage of Phi29 DNA polymerase, which produces high fidelity during DNA replication due to its proofreading 3′–5′ exonuclease activity. The reported error rate of Phi29 is between 3×10^−6^ [22] to 5×10^−6^ [23]. The probability to generate the same artifact mutation in two rounds of WGA is 9×10^−12^ to 2.5×10^−11^. In our CELSR1 mutation screen, we sequenced 192 spina bifida cases with 9045 nucleotides in the, *CELSR1*(NM_014246) gene. In total, we screened 1.74×10^6^ base pairs. The probability to detect the same coding region artifact mutation in our study is less than 4.35×10^−5^.

### Plasmids

Mouse *Ceslr1* cDNA cloned into a pEGFP-N1 plasmid (pEGFPN1-Celsr1) was kindly provided to us by Dr. Elaine Fuchs (The Rockefeller University, New York, USA). *Celsr1* open reading frame (ORF) was sub-cloned to pEGFP-C1. Human influenza hemagglutinin (HA) tagged *VANLG2* (HA-VANGL2) plasmid was obtained from Dr. Hongyan Wang (Fudan University, Shanghai, China). *VANGL2* ORF was sub-cloned into the pDs-RedC1 at XhoI and SalI restriction sites. *CELSR1* nonsense and missense changes were introduced into pEGFPN1-Celsr1 by QuikChange II Site-Directed Mutagenesis Kits (Agilent Technologies, Inc.CA,USA). All plasmids were validated by sequencing analyses.

### Subcellular localization

MDCK II cells were purchased from Sigma-Aldrich and cultured according to the manufacturer's protocols. One day before transfection, cells were seeded in 4 chamber 35 mm glass bottom dishes (4×104/chamber) (In Vitro Scientific, Sunnyvale, CA). Plasmids transfection was performed using GeneTran III Tranfection Reagent (Biomiga, San Diego, CA) according to the manufacturer's manual. Forty eight hours later, cells were washed twice with PBS and incubated 5 minutes with Hochest 3342 (1 ug/ml) (Invitrogen), then washed 3 times with PBS and fixed in 4% PFA (paraformaldehyde in phosphate-buffered saline) for 10 minutes at 37°C, followed by 3 times PBS wash. Cells were examined and photographed by an LSM710 laser scanning confocal microscope (Leica).

### Immunoprecipitation and immunoblotting

HEK293T cells were grown and maintained in DMEM supplemented with 10% fetal bovine serum (FBS) on a 6-well plate at a concentration of 6×10^5^cell/well before the day of transfection. 2 µg of GFP-Celsr1 or its related mutant plasmids were co-transfected with 2 µg of HA-Vangl2 by Lipofectamine reagent (Invitrogen). Twenty-four hours post-transfection, cells were washed twice with ice-cold PBS and lysed with 300 µl lysis buffer. Lysate was pretreated with protein A/G agarose, and then immunoprecipitated with 1–2 µg anti-HA antibody and protein A/G agarose at 4°C overnight. After washing three times with lysis buffer, the precipitates were run on SDS-PAGE followed by Western blot detection immunoblotting with the anti-GFP antibody.

## Results

### Novel rare mutations identified in *CELSR1* among spina bifida patients

**Figure 1:**
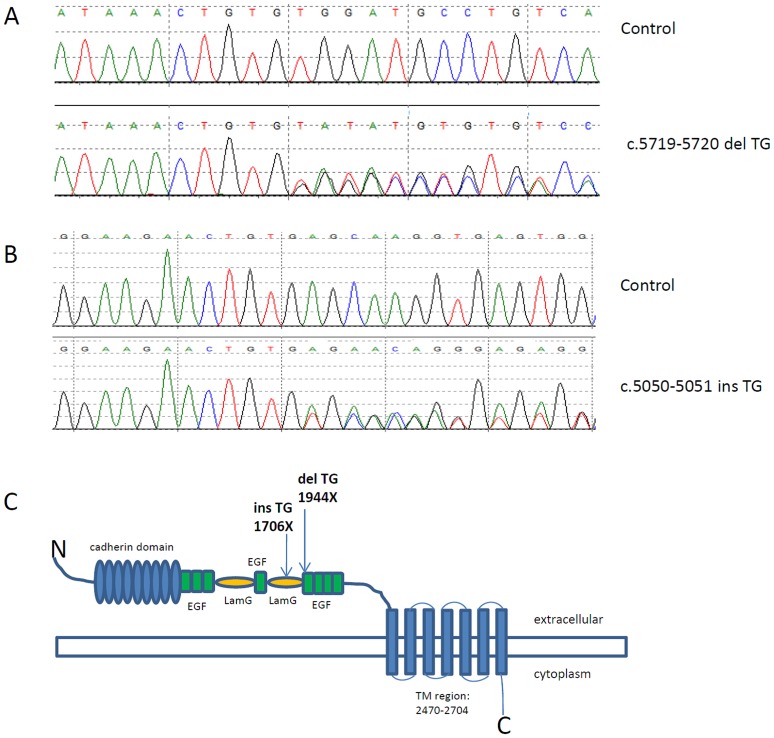
TG dinucleotide repeats variants in spina bifida. A: Sequence trace of control (top) and C.5719–5720delTG (bottom). B: Sequence trace of control (top) and TG duplication (bottom). C: Schematic representation of the CELSR1 predicted protein structure (accession number Q9NYQ6) with the domains and approximate position of TG repeats variants.

The human *CELSR1* gene coding region sequence contains 46 TGTG and 3 TGTGTG dinucleotide repeats (Figure S1). Two TG dinucleotide repeat variants were identified in spina bifida cases (N = 192), and both of them were absent among the 190 control samples (Table 1 and Figure S2). One was a TG-insertion (c.5050–5051insTG) and one was a TG-deletion (c.5719–5720delTG), both of which created a stop codon in the middle of the *CELSR1* ORF. The C.5050–5051insTG created a stop codon at the 1706^th^ amino acid, and the C.5719–5720delTG produced a stop codon at the 1944^th^ amino acid (Figure 1). We also identified 11 missense SNVs in NTD samples but not in any controls, six of which were predicted to be deleterious or damaging by both SIFT and PolyPhen (Table 1). The six mutations are p.Arg2497Cys, p.Arg2354Cys, p.Gly1410Arg, p.Thr1362Met, p.Ile1124Met and p.Ala1023Gly. Four of the 6 SNVs were predicted to be damaging and disease causing by MutationTaster and FATHMM, they were p.Arg2497Cys, p.Thr1362Met, p.Ile1124Met and p.Ala1023Gly. These four mutations were mapped to different domains of CELSR1: p.Ala1023Gly and p.Ile1124 were mapped to the cadherin repeats, p.Thr1362Met was mapped to a EGF-like domain and p.Arg2497Cye was mapped to the transmembrane domain of CELSR1 (Table 1). Among these four mutations, one (p.Arg2497Cys) was identified once in the 1000 genome sequencing project. None of the 11 SNVs were previously detected in the Exome Variants Project (Table 1) or the two previously published NTDs' CELSR1 mutation screen studies [19], [20]. All rare mutations identified in this study have been uploaded to LOVD website (http://www.lovd.nl/3.0/home).

**Table 1:** CELSR1 rare nonsynonymous variants detected in NTDs but not in controls.

Nucleotide change	rs ID	aa change	PolyPhen	SIFT	Mutation Taster	FATHMM^a^	EVP^b^	1KGP^c^	Phenotype	Domain
c.3068C>G	NA^d^	p.Ala1023Gly	**possibly damaging**	**DAMAGING**	**disease causing**	**DAMAGING**	**N.D.** e	**N.D.**	MMC^f^	Protocadherin repeats
c.3372C>G	NA	p.Ile1124Met	**possibly damaging**	**DAMAGING**	**disease causing**	**DAMAGING**	N.D.	N.D.	MMC	Protocadherin repeats
c.4085C>T	NA	p.Thr1362Met	**probably damaging**	**DAMAGING**	**disease causing**	**DAMAGING**	N.D.	N.D.	MMC	EGF-like(1–3)
c.4228G>A	NA	p.Gly1410Arg	**probably damaging**	**DAMAGING**	**disease causing**	TOLERATED	N.D.	N.D.	MMC	EGF-like(1–3)
c.4927C>T	NA	p.Arg1643Trp	benign	TOLERATED	polymorphism	TOLERATED	N.D.	N.D.	MMC	Laminin G-like 1
c.5050_5051 ins GT	NA	Truncated protein	NA	NA	**disease causing**	NA	N.D.	N.D.	MMC	Extracellular
c.5461G>T	NA	p.Val1821Leu	benign	TOLERATED	polymorphism	TOLERATED	N.D.	N.D.	MMC	Laminin G-like 2
c.5473G>A	NA	p.Gly1825Ser	benign	TOLERATED	**disease causing**	**DAMAGING**	N.D.	N.D.	MMC	Laminin G-like 2
c.5719_5720 del TG	NA	Truncated protein	NA	NA	**disease causing**	NA	N.D.	N.D.	MMC	Extracellular
c.6184G>A	NA	p.Gly2062Ser	**possibly damaging**	TOLERATED	**disease causing**	TOLERATED	N.D.	N.D.	MMC	Extracellular
c.7060C>T	NA	p.Arg2354Cys	**probably damaging**	**DAMAGING**	polymorphism	**DAMAGING**	N.D.	N.D.	MMC	Extracellular
c.7489C>T	rs200072284	p.Arg2497Cys	**probably damaging**	**DAMAGING**	**disease causing**	**DAMAGING**	N.D.	1/1000	MMC	Transmembrane
c.8632G>A	NA	p.Gly2878Ser	benign	TOLERATED	polymorphism	TOLERATED	N.D.	N.D.	MMC	Cytoplasmic domain

a We chose unweighted model for our mutation functional effect prediction;

b Exome Variants Project data (http://evs.gs.washington.edu/EVS/);

c 1000 genome sequencing data (http://www.1000genomes.org/);

d NA stands for Not available;

e N.D. indicates Not detected;

f MMC: myelomeningocele.

### The *CELSR1* C.5050–5051insTG and the C.5719–5720delTG disrupt CELSR1 membrane localization

**Figure 2:**
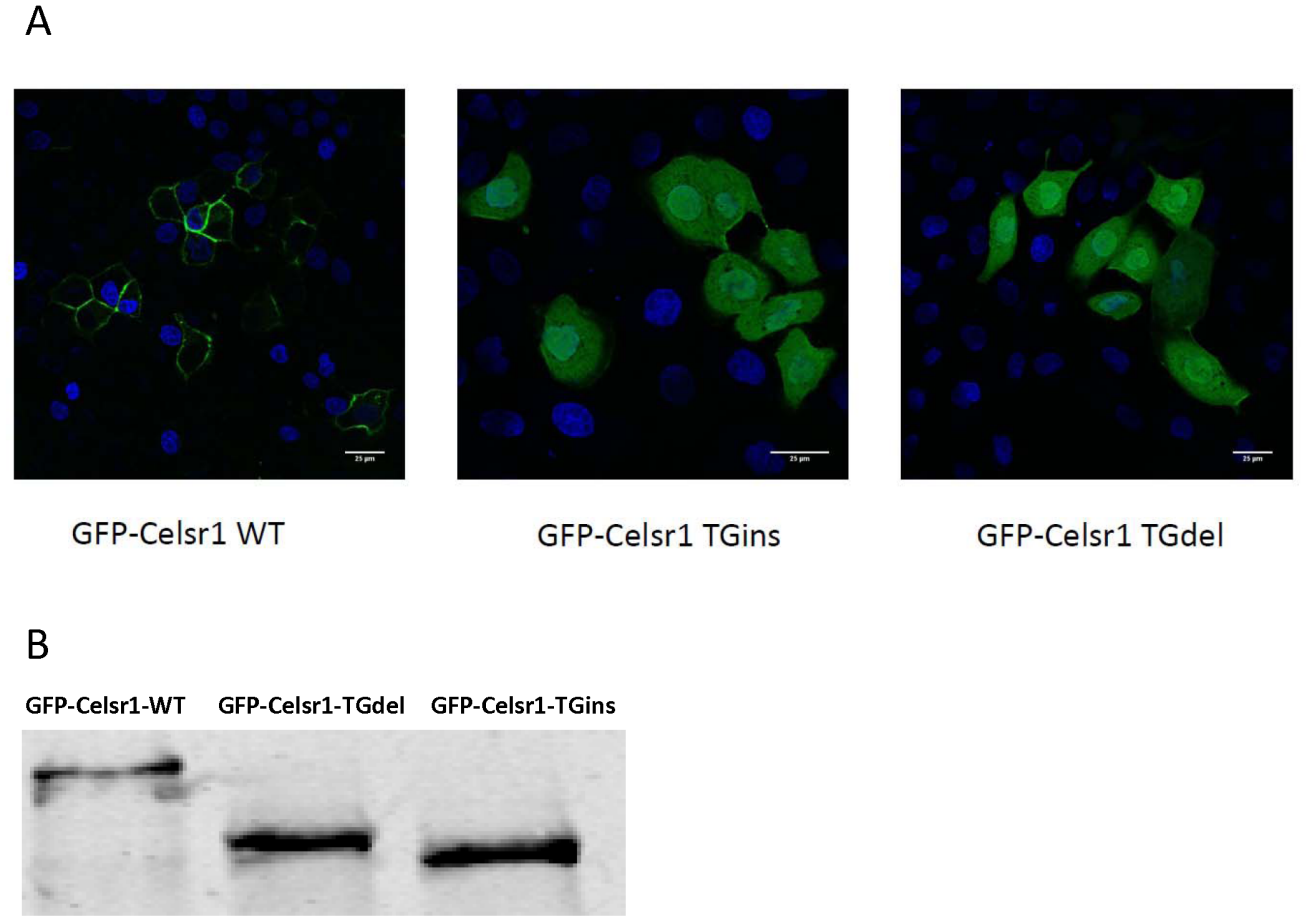
Subcellular localization of GFP-Celsr1 wild type and TG repeat variants. A: MDCK II cells were transfected with GFP-Celsr1 plasmids. Each image shown is representative of at least 50 examples. It demonstrated that the C.5719–5720delTG and the C.5050–5051insTG disrupt GFP- Celsr1 membrane localization. Scale bar, 25 µm. B: Western blot of GFP-Celsr1 wild type and the two indels mutants. It showed that the c.5719–5720delTG and the c.5050–5051insTG changed the size of GFP-Celsr1 protein.

Several point mutations (p.Ala773Val, p.Arg2438Gln, p,Ser2964Leu and p.Pro2983Ala) identified in humans craniorachischisis cases were found to alter membrane localization [19]. In our study, both the C.5050–5051insTG and the C.5719–5720delTG variantsintroduced stop codons ahead of the CELSR1 transmembrane domain. Therefore, we predicted that these two variants would also impair CELSR1 membrane localization. In the CELSR1 subcellular localization assay, both the C.5050–5051insTG and the C.5719–5720delTG misplaced CELSR1 in MDCK II cells. Unlike wild type CELSR1 which localizes almost exclusively on the cell membrane, CELSR1 protein with C.5050–5051insTG or C.5719–5720delTG distributed throughout the cells, including the cytoplasm and the nucleus (Figure 2) in all of the cells examined (100%).

### The CELSR1 C.5050–5051insTG and the C.5719–5720delTG disrupt VANGL2 cell-contacts localization

**Figure 3:**
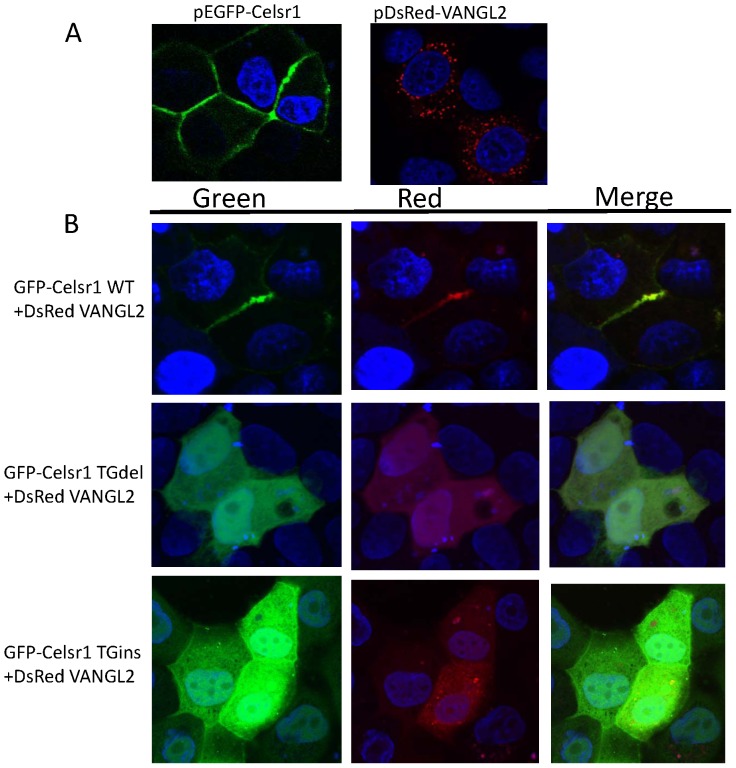
Subcellular localization of Celsr1 and VANGL2 in MDCK II cells. A: The cells were transfected with GFP-Celsr1 or DsRed-VANGL2. GFP-Celsr1 itself was localized to cell membrane while DsRed-VANGL2 was unable to localize to cell membrane. B: The cells were co-transfected with dsRed-VANGL2 (Red) and GFP-Celsr1 wild type, C.5719–5720delTG, C.5050–5051insTG. In the presence of Celsr1WT–GFP, DsRed–Vangl2 changed its localization to cell–cell borders only when two Celsr1-expressing cells are in direct contact. Each image shown is representative of at least 30 examples. It demonstrated that CELSR1 C.5050–5051insTG and C.5719–5720delTG mutants failed to recruit DsRed-VANGL2 to cell-cell contact.

It was demonstrated that Celsr1 is required to recruit Vangl2 to sites of cell-cell contacts [24]. We subsequently studied whether the C.5050–5051insTG and the C.5719–5720delTG affect the ability of CELSR1 to recruit VANGL2 to the cell-cell contact. MDCK II cells were transfected with PDS-RedC1-VANGL2 and GFP-CELSR1 wild type or its variants. CELSR1 and VANGL2 subcellular localization were examined by confocal microscopy. As shown in Figure 3, wild type CELSR1 co-localized with VANGL2 at the cell-cell contact in all of the cells examined (100%), while TG indelmutant forms of CELSR1 could not recruit VANGL2 to the contacting interface between co-transfected cells.

### CELSR1 C.5050–5051insTG and C.5719–5720delTG impair interaction with VANGL2

**Figure 4:**
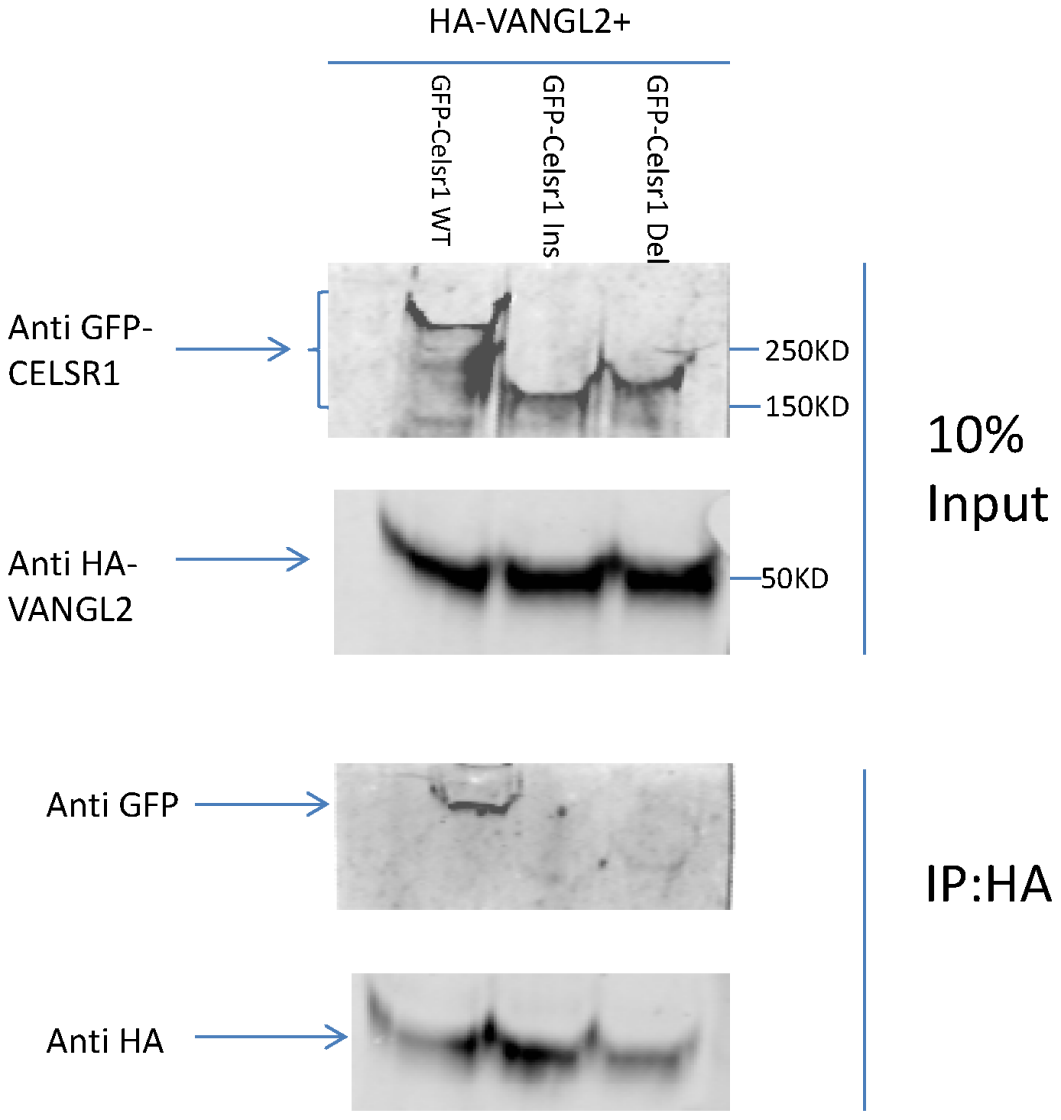
Effect of TG repeat variants on Celsr1-VANGL2 interactions by Co-Ip. Western blotting of the lysate with anti-GFP demonstrates the presence of wild-type GFP-Celsr1 protein and mutant proteins with different TG repeats. Following HA immunoprecipitation, blotting with anti-GFP confirmed physical association between Celsr1 wild type and VANGL2, while this association was affected in CELSR1 TG repeat variants.

We also tested whether the CELSR1 C.5050–5051insTG and the C.5719–5720delTG affect the interaction between CELSR1 and VANGL2. HEK293T cells were co-transfected with HA-VANGL2 and either wild type or mutant GFP-CELSR1, incubated for 24 hrs and protein lysates were subjected to co-immunoprecipitation (Co-IP) and western blot analysis. The expression levels of mutant and wild type GFP-CELSR1 were found to be similar. Co-IP assay demonstrated that C.5050–5051insTG and C.5719–5720delTG forms of CELSR1 impaired Celsr1 interaction with HA-VANGL2 (Figure 4).

## Discussion

Our study identified novel *CELSR1* TG indels and SNV's in spina bifida patients. Two previous studies reported that rare mutations in *CELSR1* are associated with human NTDs [19], [20]. Both previous studies identified only SNVs. One study investigated the biological effects of some other SNV's in detail, so our study focused on evaluating the biological effect of the observed TG repeat mutations.

Both the deletion and insertion were identified to be TG dinucleotide repeats. The insertion c.5050–5051insTG added a TG dinucleotide to TGTG, whereas c.5719–5720delTG removed a TG dinucleotide repeat from TGTGTG. It is known that among di-nucleotides, (TG)n are the most frequent in both humans and mice [25]. In humans, *CELSR1* coding sequence region (CDS) has 46 TGTG and 3 TGTGTG. In mice, *Celsr1* CDS region has 63 TGTG and 6 TGTGTG repeats. In this California spina bifida cohort, the *CELSR1* CDS TG dinucleotide repeat variation rate was 1% (2 in 192).

Previous studies showed that missense mutations in membrane associated PCP genes including *CELSR1* could affect PCP pathway signaling by disrupting membrane localization [19]. Here, micro insertions/deletions created truncated CELSR1 forms lacking the transmembrane domain, so that membrane localization might be prevented. Indeed, absence of mutant CELSR1 from the membrane and its accumulation in the cytoplasm and nuclear compartments was observed.

CELSR1 is known to physically associate with VANGL2, and CELSR1-VANGL2 interaction plays an important role in maintaining planar cell polarity during cell proliferation. During mitosis, CELSR1 interacts with VANGL2 and recruits VANGL2 to endosomes. Following mitosis, PCP proteins are recycled to the cell surface, where asymmetry is re-established by a process reliant on neighboring PCP [16]. Mutations affecting CELSR1-VANGL2 interactions can preclude VANGL2 recruitment to the endosomes during mitosis, thus disrupting PCP signaling. Both of the TG dinucleotide repeat variants identified in this study prevented Celsr1 physical association with VANGL2.

VANGL2 is another core PCP protein. It is a four-pass transmembrane protein and its proper localization is critical for VANGL2 function. Several proteins are required to establish VANGL2 localization. Previous studies demonstrated that NTD-inducing SNVs in *Vangl2* itself, such as p.D255E and p.S464N, could lead to mislocation of the protein in mice [26], [27]. SEC24B is a transport protein involved in vesicle trafficking, including shaping of the vesicle, cargo selection and concentration. Mutations of SEC24B affected VANGL2 membrane localization [28], [29]. Here, we demonstrated that truncation of CELSR1 can disrupt VANGL2 cell-cell contact localization. Our results are consistent with the findings in the previous study by Devenport and Fuchs (2008) [24], which showed that deletion of the cytoplasmic tail of GFP-Celsr1 partially impaired its localization to contacting interfaces and its ability to recruit Vangl2 at these cell-cell contacts. In our *in vivo* study, when GFP-Celsr1 wildtype was transfected alone, Celsr1 was distributed uniformly at membrane junctions (Figure 3A). When GFP-Celsr1 was co-transfected with DsRedC1-VANGL2, it was distributed asymmetrically to cell-cell contacts (Figure 3B). This observation is consistant with the Devenport and Fuchs (2008) study [24], which indicated that Vangl2 and Celsr1 are dependent on one another for their proper asymmetric distribution.

In summary, our study on a California spina bifida cohort indicates that mutations in *CELSR1* contribute to the development of spina bifida. About 1% (2 in 192) of the spina bifida cases presented TG indels in *CELSR1*. It is interesting that the two TG indels identified in this study caused severe biological malfunction of the mutant CELSR1 proteins, yet the birth defect associated with them is myelomeningocele. In contrast, the study by Robinson and co-workers [19] found SNV's that moderately altered the biological function of the CELSR1 protein, yet the associated defect was craniorachischisis, a more severe type of NTD. One possible explanation is that NTDs are caused by a combination of multiple genetic and environment factors. One functional mutation is not sufficient to produce an NTD phenotype. There likely needs to be multiple functional mutations working together to cause an NTD phenotype, and to determine the NTD subtype. It was demonstrated in mice that homozygous PCP mutations caused craniochisichisis, as shown for Vangl2, Celsr1, and Ptk7 while double heterozygosity for a PCP mutation and non-PCP mutation can cause spina bifida, as shown for Vangl2/Dact1 double heterozygotes. Perhaps the CELSR1 TG indels combined with other non-PCP mutations caused the myelomeningoceles that we observed, and that in the Robinson's study, the moderate CELSR1 mutations combined with other functional PCP mutations resulted in craniorachischisis. In our study, none of the CELSR1 TG indels were combined with mutations in other sequenced PCP genes including VANGL1, VANGL2, DISHEVELLED1,DISHEVELLED2, DISHEVELLED3, FZD6, SCRIB and PTK7. Exome sequencing or whole genome sequencing approaches are warranted in order to examine the role of additional PCP genes such as *PRICKLE*, *ANKRD6* (ankyrin repeat domain 6; also known as *DIVERSIN*, the orthologue of *diego*), *FUZ*, and to detect non-PCP genes that may contribute to these NTD phenotypes.

The four SNVs, which were predicted to be damaging by the four computational programs used in this study, were mapped to three different domains of CELSR1. Two of them, p.Ala1023Gly and p.Ile1124Met were mapped to the eighth cadherin repeat domain of CELSR1, the same domain where two previously identified NTD mouse mutations (p.Asp1040Gly and p.Asp1110Lys) were located. These two SNVs may affect the function of cadherin repeat domain which plays an important role in cell-cell contact. Variant p.Thr1362Met was predicted to change a threonine amino acid to a methionine amino acid. Thr1362 is a putative phosphorylation site of both protein kinase B (PKB) and G protein-coupled receptor kinase (GRK) based on analysis using KinasePhos 2.0 (http://kinasephos2.mbc.nctu.edu.tw/). Variant p.Arg2497Cys was mapped to the seven transmembrane domain. The amino acid characteristics change from arginine's basic to cysteine's hydroxyl may affect protein structure and CELSR1 membrane localization.

Combined with the SNVs, about 3% (6 in 192) of the spina bifida patients in our cohort possess *CELSR1* deleterious or predicted-to-be-deleterious variants. Our data provides further evidence emphasizing the contributions of PCP genes to the etiology of NTDs.

## Supporting Information

Figure S1 **The 46 TGTG and 3 TGTGTG in human CELSR1 (NM_014246) coding region sequence.** TGTG repeats were highlighted by green color and TGTGTG repeats were highlighted by red color. Both of the two TG repeat variants (c.5050–5051insTG and c.5719–5720delTG) were underlined.
(DOCX)

Figure S2 **Electropherograms of CELSR1 TG indels from genomic DNA.** Panel A indicated forward and reverse primer sequencing result of c.5719–5720del TG. Panel B indicated forward and reverse primer sequencing result of c.5050–5051ins TG.
(DOCX)
