# Supplementary material for: Identification of Novel CELSR1 Mutations in Spina Bifida
Source: PLoS One. 2014 Mar 14;9(3):e92207. doi: 10.1371/journal.pone.0092207 (PMC3954890; doi:10.1371/journal.pone.0092207)
Supplement: Figure S1 — The 46 TGTG and 3 TGTGTG in human CELSR1 (NM_014246) coding region sequence. TGTG repeats were highlighted by green color and TGTGTG repeats were highlighted by red color. Both of the two TG repeat variants (c.5050–5051insTG and c.5719–5720delTG) were underlined. (DOCX) [file pone.0092207.s001.docx]

ATGGCGCCGCCGCCGCCGCCCGTGCTGCCCGTGCTGCTGCTCCTGGCCGCCGCCGCCGCCCTGCCGGCGATGGGGCTGCGAGCGGCCGCCTGGGAGCCGCGCGTACCCGGCGGGACCCGCGCCTTCGCCCTCCGGCCCGGCTGTACCTACGCGGTGGGCGCCGCTTGCACGCCCCGGGCGCCGCGGGAGCTGCTGGACGTGGGCCGCGATGGGCGGCTGGCAGGACGTCGGCGCGTCTCGGGCGCGGGGCGCCCGCTGCCGCTGCAAGTCCGCTTGGTGGCCCGCAGTGCCCCGACGGCGCTGAGCCGCCGCCTGCGGGCGCGCACGCACCTTCCCGGCTGCGGAGCCCGTGCCCGGCTCTGCGGAACCGGTGCCCGGCTCTGCGGGGCGCTCTGCTTCCCCGTCCCCGGCGGCTGCGCGGCCGCGCAGCATTCGGCGCTCGCAGCTCCGACCACCTTACCCGCCTGCCGCTGCCCGCCGCGCCCCAGGCCCCGCTGTCCCGGCCGTCCCATCTGCCTGCCGCCGGGCGGCTCGGTCCGCCTGCGTCTGCTGTGCGCCCTGCGGCGCGCGGCTGGCGCCGTCCGGGTGGGACTGGCGCTGGAGGCCGCCACCGCGGGGACGCCCTCCGCGTCGCCATCCCCATCGCCGCCCCTGCCGCCGAACTTGCCCGAAGCCCGGGCGGGGCCGGCGCGACGGGCCCGGCGGGGCACGAGCGGCAGAGGGAGCCTGAAGTTTCCGATGCCCAACTACCAGGTGGCGTTGTTTGAGAACGAACCGGCGGGCACCCTCATCCTCCAGCTGCACGCGCACTACACCATCGAGGGCGAGGAGGAGCGCGTGAGCTATTACATGGAGGGGCTGTTCGACGAGCGCTCCCGGGGCTACTTCCGAATCGACTCTGCCACGGGCGCCGTGAGCACGGACAGCGTACTGGACCGCGAGACCAAGGAGACGCACGTCCTCAGGGTGAAAGCCGTGGACTACAGTACGCCGCCGCGCTCGGCCACCACCTACATCACTGTCTTGGTCAAAGACACCAACGACCACAGCCCGGTCTTCGAGCAGTCGGAGTACCGCGAGCGCGTGCGGGAGAACCTGGAGGTGGGCTACGAGGTGCTGACCATCCGCGCCAGCGACCGCGACTCGCCCATCAACGCCAACTTGCGTTACCGCGTGTTGGGGGGCGCGTGGGACGTCTTCCAGCTCAACGAGAGCTCTGGCGTGGTGAGCACACGGGCGGTGCTGGACCGGGAGGAGGCGGCCGAGTACCAGCTCCTGGTGGAGGCCAACGACCAGGGGCGCAATCCGGGCCCGCTCAGTGCCACGGCCACCGTGTACATCGAGGTGGAGGACGAGAACGACAACTACCCCCAGTTCAGCGAGCAGAACTACGTGGTCCAGGTGCCCGAGGACGTGGGGCTCAACACGGCTGTGCTGCGAGTGCAGGCCACGGACCGGGACCAGGGCCAGAACGCGGCCATTCACTACAGCATCCTCAGCGGGAACGTGGCCGGCCAGTTCTACCTGCACTCGCTGAGCGGGATCCTGGATGTGATCAACCCCTTGGATTTCGAGGATGTCCAGAAATACTCGCTGAGCATTAAGGCCCAGGATGGGGGCCGGCCCCCGCTCATCAATTCTTCAGGGGTGGTGTCTGTGCAGGTGCTGGATGTCAACGACAACGAGCCTATCTTTGTGAGCAGCCCCTTCCAGGCCACGGTGCTGGAGAATGTGCCCCTGGGCTACCCCGTGGTGCACATTCAGGCGGTGGACGCGGACTCTGGAGAGAACGCCCGGCTGCACTATCGCCTGGTGGACACGGCCTCCACCTTTCTGGGGGGCGGCAGCGCTGGGCCTAAGAATCCTGCCCCCACCCCTGACTTCCCCTTCCAGATCCACAACAGCTCCGGTTGGATCACAGTGTGTGCCGAGCTGGACCGCGAGGAGGTGGAGCACTACAGCTTCGGGGTGGAGGCGGTGGACCACGGCTCGCCCCCCATGAGCTCCTCCACCAGCGTGTCCATCACGGTGCTGGACGTGAATGACAACGACCCGGTGTTCACGCAGCCCACCTACGAGCTTCGTCTGAATGAGGATGCGGCCGTGGGGAGCAGCGTGCTGACCCTGCAGGCCCGCGACCGTGACGCCAACAGTGTGATTACCTACCAGCTCACAGGCGGCAACACCCGGAACCGCTTTGCACTCAGCAGCCAGAGAGGGGGCGGCCTCATCACCCTGGCGCTACCTCTGGACTACAAGCAGGAGCAGCAGTACGTGCTGGCGGTGACAGCATCCGACGGCACACGGTCGCACACTGCGCATGTCCTAATCAACGTCACTGATGCCAACACCCACAGGCCTGTCTTTCAGAGCTCCCATTACACAGTGAGTGTCAGTGAGGACAGGCCTGTGGGCACCTCCATTGCTACCCTCAGTGCCAACGATGAGGACACAGGAGAGAATGCCCGCATCACCTACGTGATTCAGGACCCCGTGCCGCAGTTCCGCATTGACCCCGACAGTGGCACCATGTACACCATGATGGAGCTGGACTATGAGAACCAGGTCGCCTACACGCTGACCATCATGGCCCAGGACAACGGCATCCCGCAGAAATCAGACACCACCACCCTAGAGATCCTCATCCTCGATGCCAATGACAATGCACCCCAGTTCCTGTGGGATTTCTACCAGGGTTCCATCTTTGAGGATGCTCCACCCTCGACCAGCATCCTCCAGGTCTCTGCCACGGACCGGGACTCAGGTCCCAATGGGCGTCTGCTGTACACCTTCCAGGGTGGGGACGACGGCGATGGGGACTTCTACATCGAGCCCACGTCCGGTGTGATTCGCACCCAGCGCCGGCTGGACCGGGAGAATGTGGCCGTGTACAACCTTTGGGCTCTGGCTGTGGATCGGGGCAGTCCCACTCCCCTTAGCGCCTCGGTAGAAATCCAGGTGACCATCTTGGACATTAATGACAATGCCCCCATGTTTGAGAAGGACGAACTGGAGCTGTTTGTTGAGGAGAACAACCCAGTGGGGTCGGTGGTGGCAAAGATTCGTGCTAACGACCCTGATGAAGGCCCTAATGCCCAGATCATGTATCAGATTGTGGAAGGGGACATGCGGCATTTCTTCCAGCTGGACCTGCTCAACGGGGACCTGCGTGCCATGGTGGAGCTGGACTTTGAGGTCCGGCGGGAGTATGTGCTGGTGGTGCAGGCCACGTCGGCTCCGCTGGTGAGCCGAGCCACGGTGCACATCCTTCTCGTGGACCAGAATGACAACCCGCCTGTGCTGCCCGACTTCCAGATCCTCTTCAACAACTATGTCACCAACAAGTCCAACAGTTTCCCCACCGGCGTGATCGGCTGCATCCCGGCCCATGACCCCGACGTGTCAGACAGCCTCAACTACACCTTCGTGCAGGGCAACGAGCTGCGCCTGTTGCTGCTGGACCCCGCCACGGGCGAACTGCAGCTCAGCCGCGACCTGGACAACAACCGGCCGCTGGAGGCGCTCATGGAGGTGTCTGTGTCTGATGGCATCCACAGCGTCACGGCCTTCTGCACCCTGCGTGTCACCATCATCACGGACGACATGCTGACCAACAGCATCACTGTCCGCCTGGAGAACATGTCCCAGGAGAAGTTCCTGTCCCCGCTGCTGGCCCTCTTCGTGGAGGGGGTGGCCGCCGTGCTGTCCACCACCAAGGACGACGTCTTCGTCTTCAACGTCCAGAACGACACCGACGTCAGCTCCAACATCCTGAACGTGACCTTCTCGGCGCTGCTGCCTGGCGGCGTCCGCGGCCAGTTCTTCCCGTCGGAGGACCTGCAGGAGCAGATCTACCTGAATCGGACGCTGCTGACCACCATCTCCACGCAGCGCGTGCTGCCCTTCGACGACAACATCTGCCTGCGCGAGCCCTGCGAGAACTACATGAAGTGCGTGTCCGTTCTGCGATTCGACAGCTCCGCGCCCTTCCTCAGCTCCACCACCGTGCTCTTCCGGCCCATCCACCCCATCAACGGCCTGCGCTGCCGCTGCCCGCCCGGCTTCACCGGCGACTACTGCGAGACGGAGATCGACCTCTGCTACTCCGACCCGTGCGGCGCCAACGGCCGCTGCCGCAGCCGCGAGGGCGGCTACACCTGCGAGTGCTTCGAGGACTTCACTGGAGAGCACTGTGAGGTGGATGCCCGCTCAGGCCGCTGTGCCAACGGGGTGTGCAAGAACGGGGGCACCTGCGTGAACCTGCTCATCGGCGGCTTCCACTGCGTGTGTCCTCCTGGCGAGTATGAGAGGCCCTACTGTGAGGTGACCACCAGGAGCTTCCCGCCCCAGTCCTTCGTCACCTTCCGGGGCCTGAGACAGCGCTTCCACTTCACCATCTCCCTCACGTTTGCCACTCAGGAAAGGAACGGCTTGCTTCTCTACAACGGCCGCTTCAATGAGAAGCACGACTTCATCGCCCTGGAGATCGTGGACGAGCAGGTGCAGCTCACCTTCTCTGCAGGCGAGACAACAACGACCGTGGCACCGAAGGTTCCCAGTGGTGTGAGTGACGGGCGGTGGCACTCTGTGCAGGTGCAGTACTACAACAAGCCCAATATTGGCCACCTGGGCCTGCCCCATGGGCCGTCCGGGGAAAAGATGGCCGTGGTGACAGTGGATGATTGTGACACAACCATGGCTGTGCGCTTTGGAAAGGACATCGGGAACTACAGCTGCGCTGCCCAGGGCACTCAGACCGGCTCCAAGAAGTCCCTGGATCTGACCGGCCCTCTACTCCTGGGGGGTGTCCCCAACCTGCCAGAAGACTTCCCAGTGCACAACCGGCAGTTCGTGGGCTGCATGCGGAACCTGTCAGTCGACGGCAAAAATGTGGACATGGCCGGATTCATCGCCAACAATGGCACCCGGGAAGGCTGCGCTGCTCGGAGGAACTTCTGCGATGGGAGGCGGTGTCAGAATGGAGGCACCTGTGTCAACAGGTGGAATATGTATCTGTGTGAGTGTCCACTCCGATTCGGCGGGAAGAAC**TGTGAG**CAAGCCATGCCTCACCCCCAGCTCTTCAGCGGTGAGAGCGTCGTGTCCTGGAGTGACCTGAACATCATCATCTCTGTGCCCTGGTACCTGGGGCTCATGTTCCGGACCCGGAAGGAGGACAGCGTTCTGATGGAGGCCACCAGTGGTGGGCCCACCAGCTTTCGCCTCCAGATCCTGAACAACTACCTCCAGTTTGAGGTGTCCCACGGCCCCTCCGATGTGGAGTCCGTGATGCTGTCCGGGTTGCGGGTGACCGACGGGGAGTGGCACCACCTGCTGATCGAGCTGAAGAATGTTAAGGAGGACAGTGAGATGAAGCACCTGGTCACCATGACCTTGGACTATGGGATGGACCAGAACAAGGCAGATATCGGGGGCATGCTTCCCGGGCTGACGGTAAGGAGCGTGGTGGTCGGAGGCGCCTCTGAAGACAAGGTCTCCGTGCGCCGTGGATTCCGAGGCTGCATGCAGGGAGTGAGGATGGGGGGGACGCCCACCAACGTCGCCACCCTGAACATGAACAACGCACTCAAGGTCAGGGTGAAGGACGGCTGTGATGTGGACGACCCCTGTACCTCGAGCCCCTGTCCCCCCAATAGCCGCTGCCACGACGCCTGGGAGGACTACAGCTGCGTCTGTGACAAAGGGTACCTTGGAATAAAC**TGTGTG**GATGCCTGTCACCTGAACCCCTGCGAGAACATGGGGGCCTGCGTGCGCTCCCCCGGCTCCCCGCAGGGCTACGTGTGCGAGTGTGGGCCCAGTCACTACGGGCCGTACTGTGAGAACAAACTCGACCTTCCGTGCCCCAGAGGCTGGTGGGGGAACCCCGTCTGTGGACCCTGCCACTGTGCCGTCAGCAAAGGCTTTGATCCCGACTGTAATAAGACCAACGGCCAGTGCCAATGCAAGGAGAATTACTACAAGCTCCTAGCCCAGGACACCTGTCTGCCCTGCGACTGCTTCCCCCATGGCTCCCACAGCCGCACTTGCGACATGGCCACCGGGCAGTGTGCCTGCAAGCCCGGCGTCATCGGCCGCCAGTGCAACCGCTGCGACAACCCGTTTGCCGAGGTCACCACGCTCGGCTGTGAAGTGATCTACAATGGCTGTCCCAAAGCATTTGAGGCCGGCATCTGGTGGCCACAGACCAAGTTCGGGCAGCCGGCTGCGGTGCCATGCCCTAAGGGATCCGTTGGAAATGCGGTCCGACACTGCAGCGGGGAGAAGGGCTGGCTGCCCCCAGAGCTCTTTAACTGTACCACCATCTCCTTCGTGGACCTCAGGGCCATGAATGAGAAGCTGAGCCGCAATGAGACGCAGGTGGACGGCGCCAGGGCCCTGCAGCTGGTGAGGGCGCTGCGCAGTGCTACACAGCACACGGGCACGCTCTTTGGCAATGACGTGCGCACGGCCTACCAGCTGCTGGGCCACGTCCTTCAGCACGAGAGCTGGCAGCAGGGCTTCGACCTGGCAGCCACGCAGGACGCCGACTTTCACGAGGACGTCATCCACTCGGGCAGCGCCCTCCTGGCCCCAGCCACCAGGGCGGCGTGGGAGCAGATCCAGCGGAGCGAGGGCGGCACGGCACAGCTGCTCCGGCGCCTCGAGGGCTACTTCAGCAACGTGGCACGCAACGTGCGGCGGACGTACCTGCGGCCCTTCGTCATCGTCACCGCCAACATGATTCTTGCTGTCGACATCTTTGACAAGTTCAACTTTACGGGAGCCAGGGTCCCGCGATTCGACACCATCCATGAAGAGTTCCCCAGGGAGCTGGAGTCCTCCGTCTCCTTCCCAGCCGACTTCTTCAGACCACCTGAAGAAAAAGAAGGCCCCCTGCTGAGGCCGGCTGGCCGGAGGACCACCCCGCAGACCACGCGCCCGGGGCCTGGCACCGAGAGGGAGGCCCCGATCAGCAGGCGGAGGCGACACCCTGATGACGCTGGCCAGTTCGCCGTCGCTCTGGTCATCATTTACCGCACCCTGGGGCAGCTCCTGCCCGAGCGCTACGACCCCGACCGTCGCAGCCTCCGGTTGCCTCACCGGCCCATCATTAATACCCCGATGGTGAGCACGCTGGTGTACAGCGAGGGGGCTCCGCTCCCGAGACCCCTGGAGAGGCCCGTCCTGGTGGAGTTCGCCCTGCTGGAGGTGGAGGAGCGAACCAAGCCTGTCTGCGTGTTCTGGAACCACTCCCTGGCCGTTGGTGGGACGGGAGGGTGGTCTGCCCGGGGCTGCGAGCTCCTGTCCAGGAACCGGACACATGTCGCCTGCCAGTGCAGCCACACAGCCAGCTTTGCGGTGCTCATGGATATCTCCAGGCGTGAGAACGGGGAGGTCCTGCCTCTGAAGATTGTCACCTATGCCGCTGTGTCCTTGTCACTGGCAGCCCTGCTGGTGGCCTTCGTCCTCCTGAGCCTGGTCCGCATGCTGCGCTCCAACCTGCACAGCATTCACAAGCACCTCGCCGTGGCGCTCTTCCTCTCTCAGCTGGTGTTCGTGATTGGGATCAACCAGACGGAAAACCCGTTTCTGTGCACAGTGGTTGCCATCCTCCTCCACTACATCTACATGAGCACCTTTGCCTGGACCCTCGTGGAGAGCCTGCATGTCTACCGCATGCTGACCGAGGTGCGCAACATCGACACGGGGCCCATGCGGTTCTACTACGTCGTGGGCTGGGGCATCCCGGCCATTGTCACAGGACTGGCGGTCGGCCTGGACCCCCAGGGCTACGGGAACCCCGACTTCTGCTGGCTGTCGCTTCAAGACACCCTGATTTGGAGCTTTGCGGGGCCCATCGGAGCTGTTATAATCATCAACACAGTCACTTCTGTCCTATCTGCAAAGGTTTCCTGCCAAAGAAAGCACCATTATTATGGGAAAAAAGGGATCGTCTCCCTGCTGAGGACCGCATTCCTCCTGCTGCTGCTCATCAGCGCCACCTGGCTGCTGGGGCTGCTGGCTGTGAACCGCGATGCACTGAGCTTTCACTACCTCTTCGCCATCTTCAGCGGCTTACAGGGCCCCTTCGTCCTCCTTTTCCACTGCGTGCTCAACCAGGAGGTCCGGAAGCACCTGAAGGGCGTGCTCGGCGGGAGGAAGCTGCACCTGGAGGACTCCGCCACCACCAGGGCCACCCTGCTGACGCGCTCCCTCAACTGCAACACCACCTTCGGTGACGGGCCTGACATGCTGCGCACAGACTTGGGCGAGTCCACCGCCTCGCTGGACAGCATCGTCAGGGATGAAGGGATCCAGAAGCTCGGCGTGTCCTCTGGGCTGGTGAGGGGCAGCCACGGAGAGCCAGACGCGTCCCTCATGCCCAGGAGCTGCAAGGATCCCCCTGGCCACGATTCCGACTCAGATAGCGAGCTGTCCCTGGATGAGCAGAGCAGCTCTTACGCCTCCTCACACTCGTCAGACAGCGAGGACGATGGGGTGGGAGCTGAGGAAAAATGGGACCCGGCCAGGGGCGCCGTCCACAGCACCCCCAAAGGGGACGCTGTGGCCAACCACGTTCCGGCCGGCTGGCCCGACCAGAGCCTGGCTGAGAGTGACAGTGAGGACCCCAGCGGCAAGCCCCGCCTGAAGGTGGAGACCAAGGTCAGCGTGGAGCTGCACCGCGAGGAGCAGGGCAGTCACCGTGGAGAGTACCCCCCGGACCAGGAGAGCGGGGGCGCAGCCAGGCTTGCTAGCAGCCAGCCCCCAGAGCAGAGGAAAGGCATCTTGAAAAATAAAGTCACCTACCCGCCGCCGCTGACGCTGACGGAGCAGACGCTGAAGGGCCGGCTCCGGGAGAAGCTGGCCGACTGTGAGCAGAGCCCCACATCCTCGCGCACGTCTTCCCTGGGCTCTGGCGGCCCCGACTGCGCCATCACAGTCAAGAGCCCTGGGAGGGAGCCGGGGCGTGACCACCTCAACGGGGTGGCCATGAATGTGCGCACTGGGAGCGCCCAGGCCGATGGCTCCGACTCTGAGAAACCGTGA

Figure S1. The 46 TGTG and 3 TGTGTG in human *CELSR1* (NM_014246) coding region sequence. TGTG repeats were highlighted by green color and TGTGTG repeats were highlighted by red color. Both of the two TG repeat variants (c.5050-5051insTG and c.5719-5720delTG) were underlined.
