# Supplementary material for: Identification of Novel CELSR1 Mutations in Spina Bifida
Source: PLoS One. 2014 Mar 14;9(3):e92207. doi: 10.1371/journal.pone.0092207 (PMC3954890; doi:10.1371/journal.pone.0092207)
Supplement: Figure S2 — Electropherograms of CELSR1 TG indels from genomic DNA. Panel A indicated forward and reverse primer sequencing result of c.5719–5720del TG. Panel B indicated forward and reverse primer sequencing result of c.5050–5051ins TG. (DOCX) [file pone.0092207.s002.docx]

 Figure S2. Electropherograms of CELSR1 TG indels from genomic DNA. Panel A indicated forward and reverse primer sequencing result of c.5719-5720del TG. Panel B indicated forward and reverse primer sequencing result of c.5050-5051ins TG.
